# Does Remote Monitoring Improve Time on Peritoneal Dialysis?: Commentary

**DOI:** 10.34067/KID.0000001086

**Published:** 2025-12-05

**Authors:** Jenny I. Shen, Tiane Dai

**Affiliations:** Division of Nephrology, Hypertension, and Transplantation, Department of Medicine, The Lundquist Institute for Biomedical Innovation at Harbor-UCLA Medical Center, Torrance, California and David Geffen School of Medicine at UCLA, Los Angeles, California

**Keywords:** dialysis, peritoneal dialysis

Many recall the era when physicians routinely made house calls, providing care in the comfort of patients' homes—a luxury that is rare today. For patients on peritoneal dialysis (PD), however, receiving treatment at home is a reality, made possible by comprehensive training for patients and their care partners. Despite the benefits of PD, in-center hemodialysis remains the preferred option for many, because of the perceived advantages of close clinical monitoring. The emergence of remote patient monitoring may shift this paradigm, offering enhanced oversight and support for patients on PD. Not only could this potentially increase the uptake and acceptance of PD, it could also prevent or delay the transition of patients to hemodialysis by preventing complications. However, multiple barriers may slow its adoption in clinical practice. In this issue, El Shamy^1^ and Perl *et al*.^2^ debate whether remote monitoring improves time on PD, highlighting both the potential and shortcomings of this technology.

The Centers for Medicare and Medicaid Services define remote patient monitoring as the use of connected medical devices that enable patients to collect and automatically transmit health data to their providers, who can then monitor and manage care remotely. In PD, remote monitoring is typically facilitated through cyclers that capture treatment parameters such as the number of completed exchanges, fill and drain volumes, dwell times, weights, BP, and alarm events. Emerging technologies even aim to detect early signs of peritonitis through effluent turbidity sensors.^3^ These data can, in theory, allow providers to help patients troubleshoot in real time, reducing unnecessary clinic visits, preventing hospitalizations by enabling earlier intervention, and alleviating patient and caregiver anxiety. By streamlining support, minimizing manual data logging, and increasing confidence in self-care, remote monitoring could feasibly extend time on PD.

Indeed, as El Shamy points out in support of remote monitoring, multiple observational studies have shown an association between remote monitoring and decreased rates of hospitalization and peritonitis as well as better sleep quality.^1,4,5^ Patients have also found it be effective and convenient.^6^ Health care providers have documented decreased costs and shorter monthly clinic visits for patients on remote monitoring.^6,7^ Most relevantly, in several studies, the advantages of remote monitoring were associated with the longer-term outcome of increased time on PD.^8,9^ Certainly, as with all observational studies, there may be confounding; it is possible that patients who use remote monitoring are also more engaged in and thus take better care of their health.

Two large randomized controlled trials of remote monitoring have been completed. Both sides of the debate cite the trial that randomized 21 hospitals (801 patients) in Mexico with automated PD programs to remote or conventional monitoring.^10^ There was no difference in the transfer to hemodialysis and the time to composite outcome #1 of all-cause mortality, first adverse event, and all-cause hospitalization. However, those on remote monitoring had a longer time to composite outcome #2 (cardiovascular mortality, first adverse event, and hospitalizations for cardiovascular disease, fluid overload, and insufficient dialysis efficiency), as well as each of the individual outcomes. Perl *et al*.^2^ also cites data from a second multicenter randomized control trial of 467 patients in Canada that found no difference in rates of transfer to hemodialysis between remote and standard monitoring.^2,11^ Thus, although remote monitoring may improve some outcomes, it has yet to be proven in a large, randomized clinical trial to increase time on therapy.

Both perspectives in this debate agree that remote monitoring's impact depends on its actual use. Currently, only patients on automated PD can benefit; those on continuous ambulatory PD lack comparable technology. Even among automated PD users, barriers such as limited Internet access and low digital literacy restrict utilization. As with most technology, cost and data privacy are concerns as well. On the provider side, access to remote data does not always translate into consistent action. Surveys have revealed that, despite broad availability to remote monitoring data, clinical teams often lack standardized protocols for reviewing and responding to information.^12^ Establishing clear workflows, including who monitors the data and how frequently, remains essential. However, these responsibilities often fall to nurses, raising concerns about additional strain on an already overextended workforce.

Both sets of authors agree that protocols for evaluating remote monitoring data and translating them into action are essential. Perl *et al*.^2^ outline potential solutions for streamlining such protocols, including better integration of the remote monitoring data into the electronic medical record. This could include dashboards that flag or highlight concerning trends in the data. Artificial intelligence could be harnessed to detect such trends and even suggest potential interventions.

Both sides of the debate acknowledge that remote monitoring has the potential to improve and extend the time on PD. The key challenge lies not in proving its potential, but in ensuring equitable, efficient, and sustainable integration into routine clinical practice.
